# CO_2_-storage assessment and effective capacity in Algeria

**DOI:** 10.1186/s40064-016-2682-7

**Published:** 2016-07-11

**Authors:** Abdelouahab Aktouf, Abdelhakim Bentellis

**Affiliations:** Houari Boumediene University of Sciences and Technology, Bab Ezzouar, Algeria

**Keywords:** Carbon capture and storage, Algeria, Basin assessment, Site screening, Storage capacity

## Abstract

Deep saline aquifers widely distributed deep in the earth offer the greatest CO_2_ storage potential in all current geological CO_2_ storage approaches. The western region of the Saharan platform in Algeria includes several sedimentary basins characterized by a large production of dry gas with high CO_2_ rates sometimes exceeding 9 %. To reduce CO_2_ emissions, these basins were analyzed to identify those with the largest potential for the geological sequestration of CO_2_ (GSC). The evaluation methodology applied to determine the basin potential is based on qualitative geological and practical criteria to which we have assigned normalized numerical values. This evaluation method allows us to quantitatively compare and evaluate the basins in Algeria. Estimations of the CO_2_ storage capacities of several structures in the sedimentary Ahnet–Gourara Basin, which has the greatest potential for GSC, vary from 1 Gt to over 5 Gt. Based on cautious estimations, these geologic structures should be able to contain the entire volume of the CO_2_ emitted over the next three decades at least.

## Background

It is widely believed that one primary reason for global warming is the rapid increase of atmospheric greenhouse gases caused by the exponential growth in the amount of CO_2_ released into the atmosphere since the industrial era. Developing CO_2_ capture and storage techniques, improving energy efficiency, increasing the use of renewable resources (e.g., solar energy), and using fossil fuels more rationally could be key to reducing atmospheric CO_2_ emissions. In 1993, Algeria ratified the Framework Convention of the United Nations on Climate Change (UNFCCC) developed at the Earth Summit in Rio in 1992 and acceded to the Kyoto Protocol in 2004, demonstrating its intent to participate in the international effort to fight climate change and its repercussions, particularly regarding the climate system, natural ecosystems, and sustainable economic development.

**Fig. 1:**
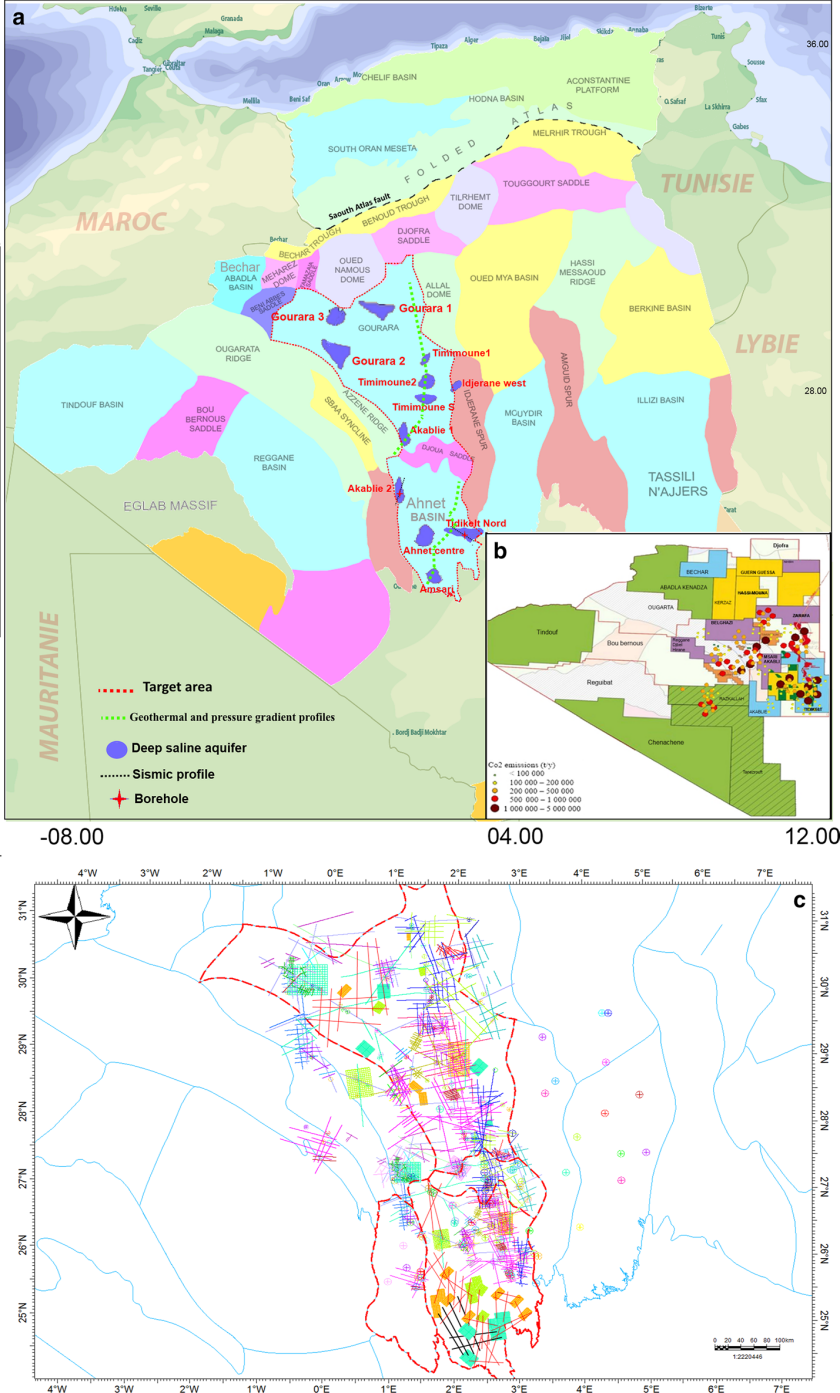
**a** Simplified geological map of Algeria (Beuf 1971) showing the location of the 12 potential areas suitable for CO_2_ geological storage (*dark blue areas*). **b** Locations of 2D seismic lines, 3D seismic survey areas and wells used for modeling. **c** The location of the major CO_2_ point sources (emissions >0.1 Mt/year) in the southwest part of Algeria; the values were calculated based on the dry gas production in the various fields

Currently, global anthropogenic emissions amount to approximately 26 Gt/year (Herzog and Golomb 2004). In Algeria, CO_2_ emissions were approximately 117,310 million tons in 2000 (PNUD 2010). As an example, different sources of CO_2_ emissions in southwest Algeria are shown in Fig. 1b based on the production of dry gas in different sites. Currently, CO_2_ capture and sequestration (CCS) has attracted interest because it represents, in the medium and short terms, a viable potential solution to reduce anthropogenic CO_2_ emissions to the atmosphere (De Connick, et al. 2005; IEA 2013 ). In fact, CO_2_ sequestration technology in gas and oil reservoirs and in deep saline aquifers is already in use. Worldwide, the search for potential sites for geological CO_2_ sequestration is underway in sedimentary basins that are known for their geological reservoir quality (Bachu et al. 2007; Bradshaw et al. 2007; Ogawa et al. 2011; Mao et al. 2014).

Southwestern Algeria, which is characterized by high dry gas production with CO_2_ levels sometimes exceeding 9 %, is divided into several sedimentary basins that were analyzed to identify those with the greatest potential for the geological sequestration of CO_2_ (GSC). This study represents the first assessment of the potential storage of this greenhouse gas in deep Algerian saline aquifers mainly using seismic and logging data.

## Geological setting

The Algerian sedimentary basins have a geological history involving the global geodynamical process of plate tectonics that structured Algeria into two areas, North Algeria and the Saharan platform, separated by the South Atlas Fault. The southwest part of the Algerian Sahara, which is the subject of this study, is geologically linked to the western part of the African slab and is limited in the south by the shield borders of the Reguibat (Egleb-Yetti) and Touareg (Hoggar) and in the north by the deep South Atlas collision zone, separating the Precambrian and the Mesozoic active epi-Hercynian platforms (Fig. 1a). Sediments from the Paleozoic geo-structural era are most ubiquitous in the Algerian Sahara; this is the reason why it serves as a reference for the division of the Sahara into tectonic regions.

The various data collected in the field during seismic campaigns helped distinguish the different tectonic phases that affected the sedimentary cover in the region, such as the Pan-African phase and the Pan-African heritage, which began 780 Ma and involved large vertical movements and calc-alkaline volcanic andesitic type “active margin” activity related to subduction phenomena (Drid 1989). Approximately 600 Ma, a continental collision occurred, corresponding to a major phase in the Pan-African orogeny. Because of this E–W compression, tightening between the two cratons (rigid West Africa and East African Nilotic) occurred with meridian folds, metamorphism, and granitization. The Algerian Sahara was subjected to these vertical movements and was accompanied by volcanic eruptions and uplifts, which eroded the sedimentary cover.

One major structural feature is the disposition in longitudinal compartments corresponding to horsts and grabens. The ridges that separate them and have WNW-SSE orientations correspond to major shears. Lateral movements can be very large and exceed hundred kilometers (Caby 1968). The most important structural elements of these basins were formed during the Hercynian Orogenic phase. The post-Hercynian Orogenic movements had little effect on their structures. The impact on the Hercynian unconformity is practically non-existent, except in a few places where slight deflections are barely detectable.

The Tassili phase at the beginning of the Cambrian is indicated by the unconformity in the Cambrian deposits on the Precambrian basement. The Taconic phase was responsible for the erosion of the Ordovician deposits. The Ardennes phase started at the end of the Silurian, and during this period, a regime change occurred in the regressive tendency with positive decreased epeirogenic movements resulting in the tilting of the basin to the south during the Silurian and to the north during the lower Devonian until the generalized marine transgression. The Late-Hercynian phase is marked by the formation of direction folds, N130° folds parallel to the Ougarta chain (Haddoum et al. 2001). Finally, an Alpine phase generated N110° folds and reactivated the N-S strike-slip fault. Haddoum et al. (2001) suggested the existence of Hercynian post-moscovian phases with a constrained NNE-SSW orientation. Therefore, the area consists of a set of folds and relays with variable extensions associated with deep faults.

## Basin evaluation

The methodology used to evaluate the sedimentary basins’ potential for GSC is based on the work of Bachu (2003) and CO2CRC (2008). This potential was determined according to several geological characteristics and practices Table 1. The methodology used in this study allows for the conversion of these qualitative characteristics to quantitatively evaluate a few specific criteria. The basin analysis was based on the set of criteria and classes listed in Table 1. In the first step, which qualitative class (j = 1, …, n) the basin belongs to for each of the fifteen evaluation criteria (i = 1, …, 15) is determined. Three to five classes (n = 3, 4, or 5) are used to evaluate each criterion. The existing geological and geophysical data and the geographic and geological knowledge of the basins are essential to properly evaluate the criteria (see next chapter). Each class has an individual value (F_i,j_), which helps transform the qualitative values (basin characteristics) into comparable quantitative values. The less and more favorable classes have the lowest and highest values, respectively. The individual value of each class is determined depending on its importance for GSC. Thus, if the classes are similarly important, the linear variation in the class values can be used. Conversely, if the most favorable classes are more important than the others, the variation in the values will be exponential. Table 2 shows the values of the various classes used in the framework of this study of the sedimentary basins in southwest Algeria. The individual values of the classes (F_i,j_) shown in Table 2 and the weights assigned to the criteria (w_i_) were modified to account for the basins’ intrinsic characteristics.

**Table 1:** Evaluation criteria of the sedimentary basins potential for the geological sequestration of CO_2_. Modified from Bachu (2003)

Criteria	Classes				
	j = 1	j = 2	j = 3	j = 4	j = 5
i = 1					
Seismicity (tectonic environment)	Very high	High	Intermediate	Weak	Very weak
i = 2					
Area	<1000 km^2^	1000–5000 km^2^	5000–25,000 km^2^	25,000–50,000 km^2^	>50,000 km^2^
i = 3					
Depth	Very deep (<300 m)	Shallow (300–800 m)	Deep (>3500 m)	Intermediate (800–3500 m)	
i = 4					
Deformation	Important	Moderate	Weak		
i = 5					
Reservoir-coverage	Weak	Intermediate	Excellent		
i = 6					
Geothermal power	Warm basin (>40 °C/km)	Moderate basin (30–40 °C/km)	Cold basin (<30 °C/km)		
i = 7					
Potential in hydrocarbons	None	Weak	Average	High	Huge
i = 8					
Evaporites	None	Domes	Beds		
i = 9					
Coal	None	Deep (> 800 m)	Shallow (200–800 m)		
i = 10					
Exploration Maturity	Not explored	Exploration	Development	Mature	Super mature
i = 11					
On/offshore	In shallow sea	In shallow sea	In shallow sea and inland	Inland	
i = 12					
Climate	Arctic	Subarctic	Desertic	Tropical	Temperate
i = 13					
Accessibility	Inaccessible	Difficult	Acceptable	Easy	
i = 14					
Infrastructure	None	Minor	Moderate	Important	
i = 15					
Sources of CO_2_	None	Little	Moderate	Significant	Several

**Table 2:** Values and weight of the criteria and classes for the evaluation of sedimentary basins in South west of Algeria for the geological sequestration of CO_2_. Modified from Bachu (2003)

Criteria	Classes					Weight (wi)
	j = 1	j = 2	j = 3	j = 4	j = 5	
i = 1						
Seismicity	1	3	7	15	15	0.1
i = 2						
Area	1	3	5	8	10	0.06
i = 3						
Depth	1	2	6	10		0.1
i = 4						
Deformation	1	4	10			0.08
i = 5						
Reservoir-coverage	1	4	10			0.1
i = 6						
Geothermal power	1	4	10			0.09
i = 7						
Potential in hydrocarbons	1	3	7	14	21	0.04
i = 8						
Evaporites	1	2	3			0.01
i = 9						
Coal	1	2	5			0.04
i = 10						
Exploration maturity	1	3	4	8	10	0.08
i = 11						
On/offshore	1	5	10	15		0.11
i = 12						
Climate	1	2	4	7	10	0.04
i = 13						
Accessibility	1	3	6	10		0.04
i = 14						
Infrastructure	1	3	7	10		0.05
i = 15						
CO_2_ sources	1	3	7	11	15	0.06

To compare the various class values of each of the evaluation criteria for sedimentary basin *k*, the individual values (F_i,j_) were normalized according to:

1 $$P_{i}^{k} = \frac{{F_{i,j} - F_{i,1} }}{{F_{i,n} - F_{i,1} }}.$$

This equation distributes the class values between 0 and 1. Therefore, for all the evaluation criteria, the least favorable class has a P_i_ value of 0, and the most favorable class has a P_i_ value of 1. Thus, each basin *k* is characterized by 15 normalized individual values.

Each criterion has a different importance in the evaluation of the basins. Therefore, a criterion with a high influence on the CO_2_-sequestration potential will have a higher weight (w_i_) than a less-significant criterion. The weights of the criteria are shown in Table 2. The weights satisfy the following condition:

2 $$\mathop \sum \limits_{i = 1}^{15} W_{i} = 1$$

The final score of each basin (R^k^) is ultimately calculated using a weighted average of the normalized individual values and the weights of the corresponding criteria:

3 $$R^{k} = \mathop \sum \limits_{i = 1}^{15} W_{i} P_{i}^{k}$$

The basins with the highest final scores have the highest potential for GSC. It is therefore possible to identify the basins that should be studied in greater detail and to proceed to evaluating specific sites for GSC.

## Evaluation of the Algerian reservoir formations suitable for GSC

The identification of potential geological storage reservoirs is one of the first steps to determining the most suitable basin for GSC. This basin must meet the following criteria (De Connick et al. 2005):

- contain excellent reservoir-cover sets;
- have abundant seismic data and wells;
- be easily accessible and have adequate infrastructure development;
- be available; and
- have saline aquifers at depth within the sedimentary sequence.

These criteria are used to calculate the basins’ final scores and objectively compare them to determine the most appropriate one for GSC. The characteristics of six sedimentary basins in southwest Algeria are summarized in Table 3.

**Table 3:** Evaluation of the criteria and ranking of the potential of southwesterern Algeria sedimentary basins for CO_2_ storage

Criteria	Sedimentary basins						Weight (wi)
	Bechar	Tindouf	Reggane	Cuvette de Sbaa	Ahnet	Gourara	
i = 1							
Seismicity	5	5	5	5	5	5	0.1
i = 2							
Area	3	5	5	3	5	5	0.06
i = 3							
Depth	2	2	4	4	4	4	0.1
i = 4							
Deformation	2	2	2	2	3	3	0.08
i = 5							
Reservoir-coverage	2	2	2	2	3	3	0.1
i = 6							
Geothermal power	3	3	2	2	2	2	0.09
i = 7							
Potential in hydrocarbons	2	2	4	4	5	5	0.04
i = 8							
Evaporites	2	2	2	2	2	2	0.01
i = 9							
Coal	1	1	1	1	1	1	0.04
i = 10							
Exploration maturity	2	2	3	3	5	5	0.08
i = 11							
On/offshore	4	4	4	4	4	4	0.11
i = 12							
Climate	3	3	3	3	3	3	0.04
i = 13							
Accessibility	2	2	3	3	4	4	0.04
i = 14							
Infrastructure	1	1	2	3	4	4	0.05
i = 15							
CO_2_ sources	1	1	3	4	5	5	0.06
	0.48	0.51	0.62	0.63	0.90	0.91	

**Fig. 2:**
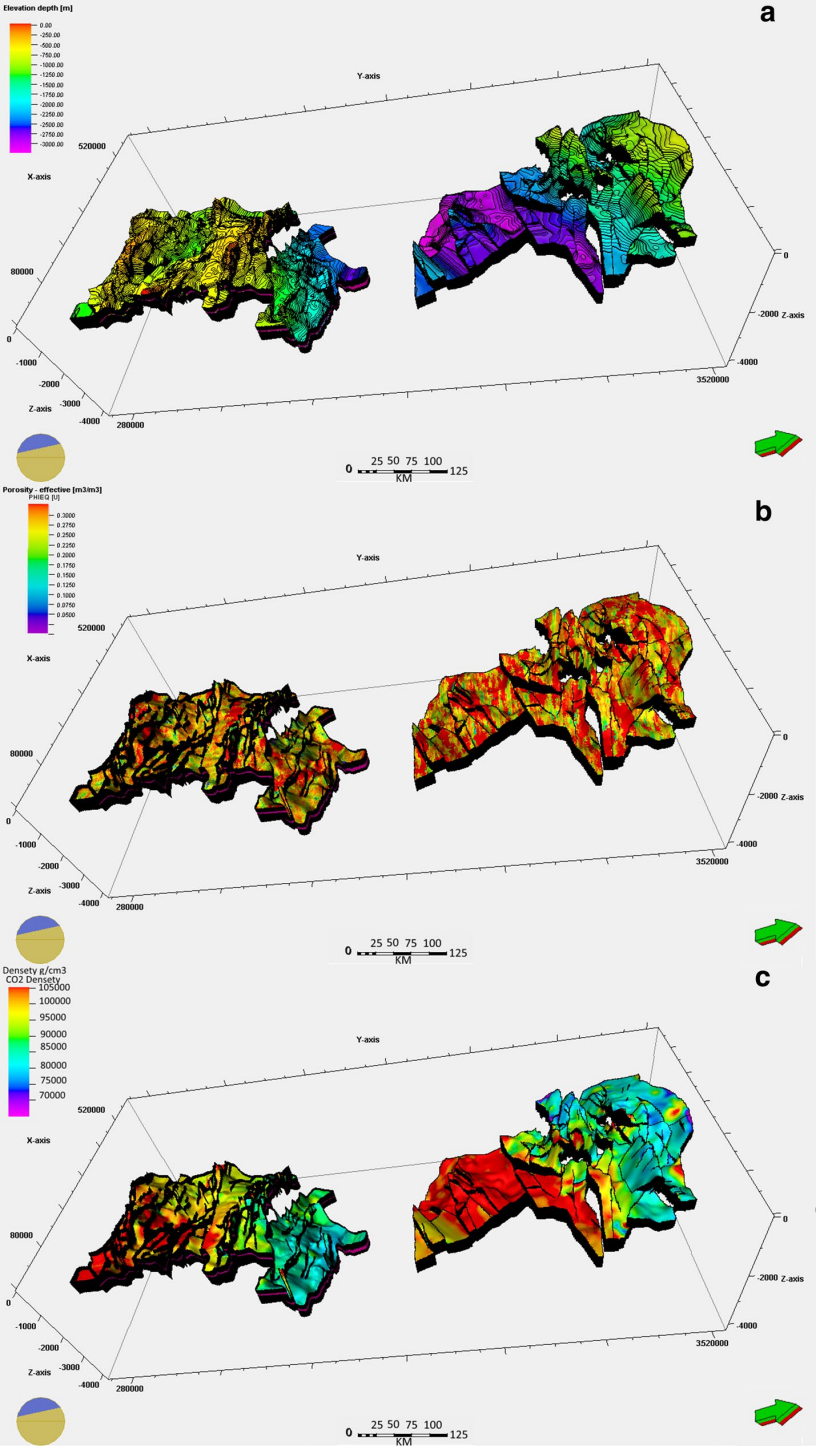
**a** Geological model of Cambro–Ordovician and Devonian reservoirs in Ahnet–Gourara. The location of this model is shown in Fig. 1a. The color indicates the surface depth of reservoirs. **b** Effective porosity distribution of Cambro–Ordovician and Devonian reservoirs in Ahnet–Gourara. **c** CO_2_ density distribution of Cambro–Ordovician and Devonian reservoirs in Ahnet–Gourara

Suitable geological formations for CO_2_ storage occur at a depth of 800–1000 m. These formations should be able to maintain the injected CO_2_ in a supercritical state, which has a liquid-like density (approximately 500–800 kg/m^3^; Fig. 2c), and thus facilitate efficient space filling of the underground storage volume. This density also allows the maintenance of low buoyancy and leads to a high CO_2_ storage capacity (De Connick et al. 2005).

To locate geological formations with these characteristics in the Ahnet–Gourara Basin in southwest Algerian, we were granted access to more than 250 wells and 45,000 km of two-dimensional (2D) seismic profiles and 8750 km^2^ of three-dimensional (3D) seismic profiles (Fig. 1c) by the National Agency for the Valorization of Hydrocarbon Resources (ALNAFT) and the National Society for Research, Production, Transportation, Processing, and Marketing of Hydrocarbons (SONATRACH) as part of a thesis project. The data have been collected by several oil companies since 1965.

The methodology used to evaluate the geological storage capacity in the Ahnet–Gourara Basin allows the effective CO_2_ storage capacity (CO_2_ mass) to be calculated according to the techno-economic pyramid resources (Bachu et al. 2007). The effective capacity is defined as a subset of the total capacity of the reservoir or the theoretical capacity, which is obtained by restricting the storage ability according to the available techniques and geological boundaries.

According to the methodology adopted by the National Atlas of Canada and the U.S. Department of Energy (2008), a basin’s effective storage capacity is limited by the minimum depth and the factor of effective storage of CO_2_ in saline aquifers. This minimum depth is 800 m, which is necessary to ensure the safety of the storage sites (Bachu et al. 2010). The efficiency factors allow us to estimate the proportion of the volume of the reservoir that could be occupied by the injected CO_2_.

The volumetric capacity calculation uses the equation below:

4 $$M_{{{\text{CO}}_{2} }} = {\text{E}}_{\text{salin}} *{\text{A}}*{\text{h}}*\Phi *\uprho_{{{\text{CO}}_{2} }} ,$$

where M_CO2_ is the effective storage capacity (tons); E_salin_ is the storage efficiency factor; A is the area that defines the basin or the region occupied by the aquifer (m^2^); h is the effective thickness, i.e., the average thickness of the aquifer × the average net-to-gross ratio (m); Φ is the average reservoir porosity (%); and $\uprho_{{{\text{CO}}_{2} }}$ is the density of the CO_2_ at reservoir conditions (kg/m^3^).

The CO_2_ storage efficiency factors E_salin_ for the saline aquifers vary according to the lithology of the reservoir units. The efficiency factors calculated for deep saline aquifers (NETL 2010) should consider the following:

- the fraction of the aquifer that can be occupied by CO_2_;
- the fraction of the unit with adequate porosity and permeability for CO_2_ injection;
- the fraction of the porosity that is interconnected;
- the efficiency of horizontal and vertical movement; and
- the efficiency of the displacement pores and CO_2_ buoyancy scale (Van der Meer and Egberts 2008).

Operational procedures (e.g., injection pressure) could further influence the injected CO_2_ behavior as well as storage efficiency factors (Tsuji et al. 2016). This factor ranges between 1 and 4 % (U.S. Department of Energy 2008). The EU Geo-Capacity Project suggested a value of 2 % as a cautious estimate of the CO_2_-storage capacity in regional saline aquifers (Vangkilde-Pedersen et al. 2008).

The reservoir area was determined after identifying the reservoirs and caprock formations by interpreting 2D and 3D seismic profiles Fig. 1c and evaluating the vertical seismic profiles (VSP) to set the horizon using the Schlumberger Petrel software. Geological modelling uses integrated data sets to determine the geological framework that best represents the major geological features that should be incorporated into a representation of a region, Special attention was also given to the structural modeling to reveal any structural characteristics or faults in the caprock-reservoir system that could represent preferential conduits for CO_2_ leakage. The first step in constructing a 3D geological model (Fig. 2a) is to build the fault network and horizon top model. Fault network and horizon tops Modelling connectivity and flow through fractured rock is a difficult and critical problem to solve for many sectors. Understanding flow through faults and joints is also important.

Rock porosity can be obtained from the sonic, density, or neutron logs. The log response is affected by the formation porosity, the fluid, and the matrix. If the fluid and matrix effects are known or can be determined, the tool response can be related to the porosity. Therefore, these devices are often referred to as porosity logs. All three logging techniques respond to the characteristics of the rock immediately adjacent to the borehole. Their depths of investigation are very shallow from the borehole wall and, therefore, generally fall within the flushed zone.

Other petro-physical measurements, such as micro resistivity, nuclear magnetism, or electromagnetic propagation, may be used to determine porosity. However, these devices are strongly influenced by the fluid saturating the rock pores.

Effective porosity distribution (*Ø*_*e*_), the volume of interconnected pores through which fluid can flow; Fig. 2b) in saline aquifers have been determined using the Schlumberger Techlog software based on the density probe gamma–gamma (density porosity *Ø*_*D*_ or overall density (*ρ*_*b*_), porosity neutron probe (*Ø*_*N*_), and natural gamma probe (GR) logs. Two different methods have been used, depending on the availability of the logging of the photoelectric factor (PEF) and the empirical method using sonic logs (Schlumberger 2000).

If PEF is present, the relative volumes of the minerals forming the rock (e.g., silica, calcite, dolomite, and shale) are known, and *ϕ*_*e*_ can then be calculated using the Doveton method (Doveton 1986, 1994):

5 $$\phi_{e} = \phi_{t} /\left( {1 - {\text{Vsh}}} \right),$$

where *ϕ*_*t*_ is the total porosity calculated from the lithology, and Vsh is the shale volume.

6 $$\phi_{t} = \, (\uprho_{m} - \uprho_{b} ) /(\uprho_{m} - \uprho_{f} ),$$

where $\uprho_{m}$ is the density of the matrix obtained from the lithology, $\uprho_{b}$ is the bulk density, and $\uprho_{f}$ is the density of the fluid present in the pores.

If photoelectric factor PEF is unavailable, *ϕ*_*e*_ is calculated as:

7 $$\phi_{e} = ((\phi_{D} + \phi_{N} )/2.0)*\left( {1 - {\text{VSH}}} \right),$$

where DPHI is the porosity obtained from the density probe, and NPHI is the porosity obtained from the neutron probe.

**Fig. 3:**
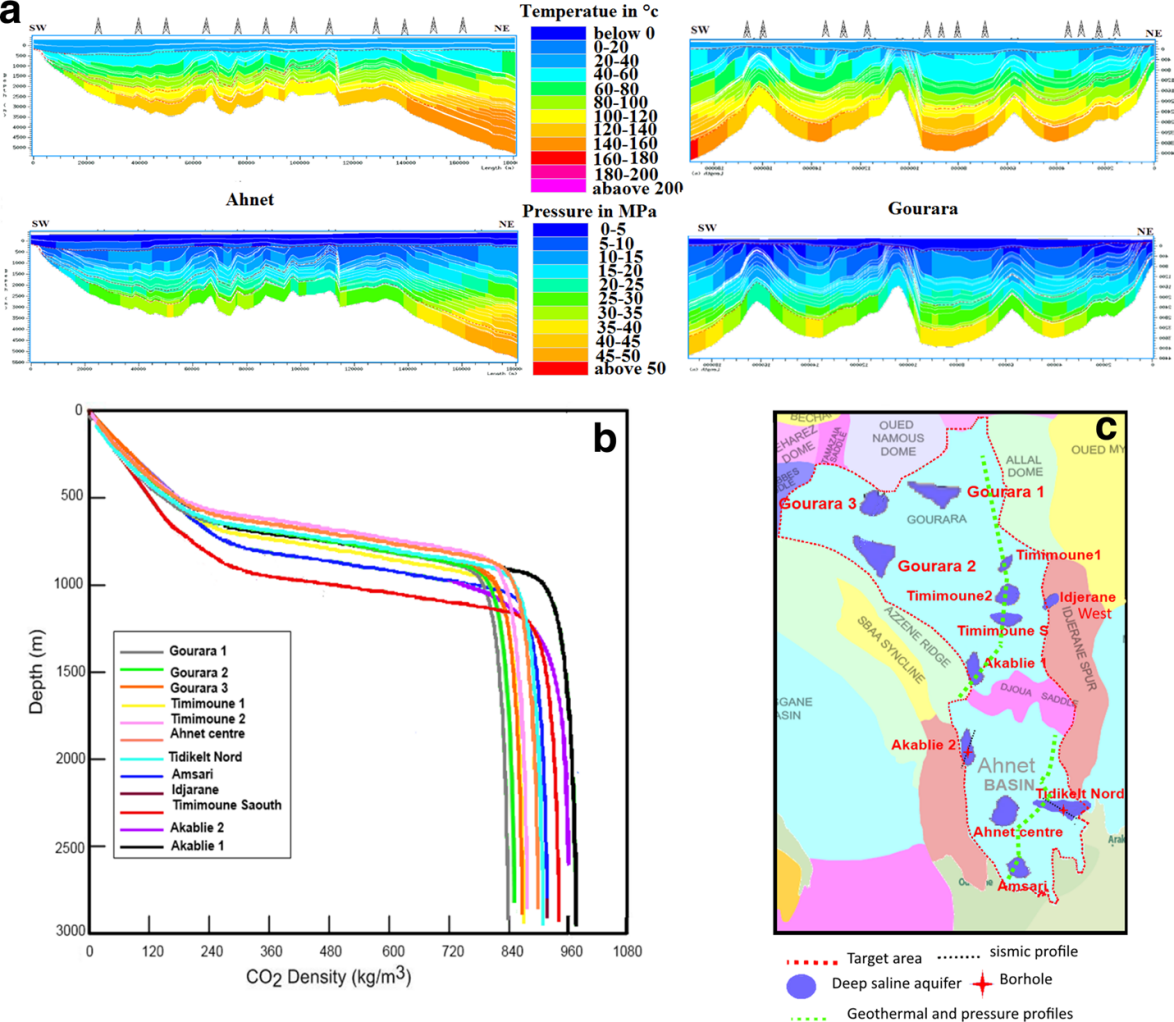
**a** Geothermal and pressure gradient of Ahnet–Gourara basins. The locations of these profiles are shown as *green dashed lines* in (**c**) and Fig. 1a . **b** CO_2_ density as a function of depth at the different potential confinement sites. **c** The location of the 12 potential areas suitable for CO_2_ geological storage (*dark blue areas*)

The CO_2_ density ($\uprho_{{{\text{CO}}_{2} }}$) at depth (kg/m^3^) varies depending on the temperature and pressure. The temperature (Fig. 3a) is defined by the equation:

8 $${\text{T}} = {\text{Ts}} + \left( {\Delta _{\text{Z}} *{\text{geothermal gradient}}} \right),$$

where T is the temperature at depth (in meters), Ts is the surface temperature (in meters), Δ_Z_ is the depth from the surface (in meters), and the geothermal gradient is calculated (°C/m).

The pore pressure is estimated by the following equation:

9 $${\text{P}} =\Delta _{Z} *{\text{pressure gradient}}*1.1,$$

Particular attention was given to these two parameters because they affect the density of CO_2_, which in turn affects the storage capacity (Bachu 2003). The CO_2_ density was calculated using a program developed at the Ruhr-Universität Bochum and based on the work of Span and Wagner (1996); the results are shown in Fig. 2c and the Fig. 3b shows Graph of the CO_2_ density as a function of depth at the different potential confinement sites.

**Fig. 4:**
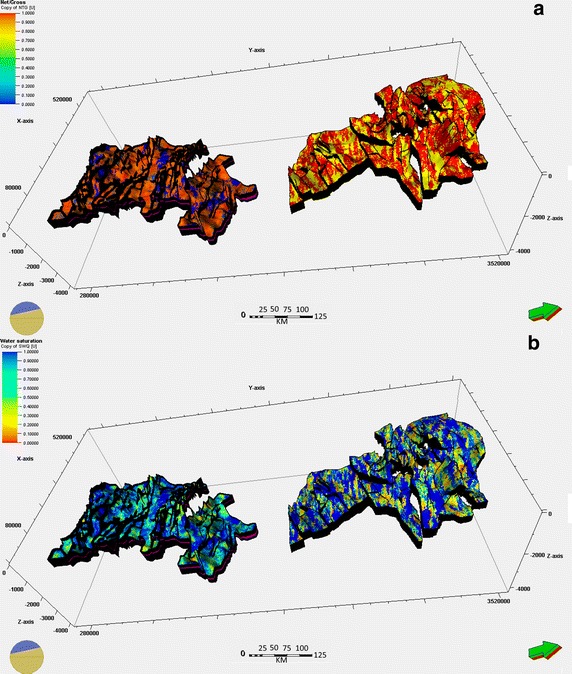
**a** Net gross model of Cambro–Ordovician and Devonian reservoirs in Ahnet–Gourara. **b** Water saturation distribution of Cambro–Ordovician and Devonian reservoirs in Ahnet–Gourara

**Fig. 5:**
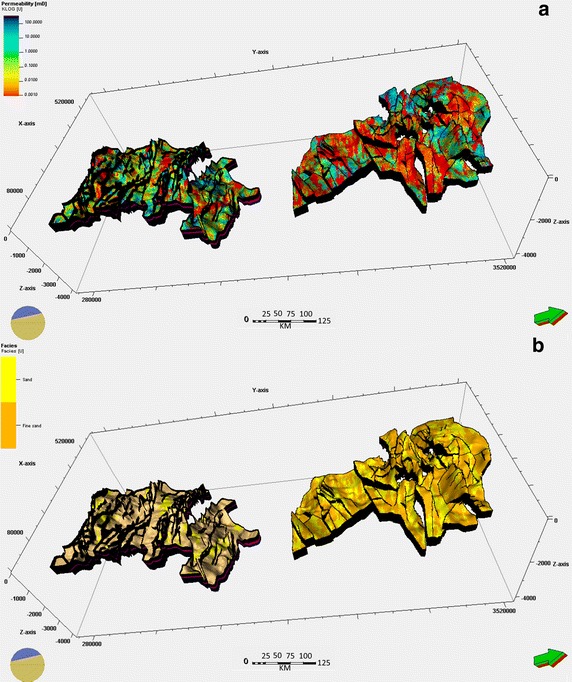
Permeability and facies distribution of Cambro–Ordovician and Devonian reservoirs in Ahnet–Gourara

The effective thickness (Fig. 4a) was calculated by considering the sum of the thicknesses of each porous permeable layer in the reservoir. A minimum threshold of porosity (the porosity cut-off) and permeability (Fig. 5a) (the permeability cut-off), K (md), was established to determine the favorable CO_2_ injection intervals and identify additional pore volumes to control the storage of CO_2_. Gamma ray (GR) measurements were used to determine the thickness of the clay intercalations.

## Site screening

This analysis was mainly focused on the geological (structural and stratigraphical) characterization of the potential reservoir–caprock systems.

The purpose of the Site Screening stage of the Exploration Phase is to evaluate sub-regional basin data sets and assess storage potential within a defined sub-region. This stage utilizes primarily existing data and resources for this assessment which classifies storage potential as Prospective Storage Resources. The initial evaluation conducted during this stage, evaluates a Potential Sub-Region through each component analysis resulting in a set of selected areas. These areas are then ranked based on criteria established during Project Definition and the highest ranking Selected Area advances to the next evaluation stage. This process is analogous to the maturation of a petroleum project from “play” to “lead”. The Site Screening evaluation performed on Potential Sub-Regions includes four components for analysis:

- Injection Formation: identify regional and sub-regional formations that have geologic characteristics that are suitable for storage.
- Adequate Depth: ensure that formations have regional extent with sufficient depth to maintain injected CO_2_ in the supercritical state.
- Confining Zone: ensure adequate confining zone is present and have lateral extent to contain injected CO_2_ and avoid vertical migration of brine into a underground source of drinking water.
- Prospective Storage Resources: calculate the prospective storage resource to ensure that formations have sufficient pore volumes and can accept the change in pressure to accommodate planned injection volumes.

A regional-level assessment of potential storage sites for CO_2_ in Ahnet–Gourrara province was carried out. This work was undertaken using a site-by-site approach to analysis the geology, storage capacity and its suitability to store CO_2_. GIS database of each basin including a lot of basic information, such as strata depth, strata thickness, structural map, was established according to seismic survey, well data and published information. These data were utilized to calculate CO_2_ storage capacity where possible.

The analyse of Ahnet–Gourrara Basin has identified 12 suitable areas, represented by laterally semi-confined to confined deep saline aquifers (Fig. 1a). The main characteristics of these potential areas are summarized in Table 4. The selected areas reveal thick accumulations of sediments, permeable rock formations saturated with saline water and extensive covers of low porosity rocks (acting as seals).

**Table 4:** Key parameters of the Ahnet–Gourara potential reservoirs for the evaluation of the CO_2_ storage capacity input data from Sonatrach

Site name	Avarage reservoir depth (m)	Area (km^2^)	Effective thickness (m)	Porosity (%)	Geothermal gradient (°C/km)	Storage capacity (Mt) Seff = 1 %	Storage capacity (Mt)
Seff = 5 %							
Akablie 1	1250	100	160	15	30	23.3	116.4
Akablie 2	1165	480	500	15	30.5	345.6	1728
Tidikelt Nord	1200	1150	250	18	30	476.1	2380.5
Ahnet centre	1300	180	120	14	31.04	27.8	139.1
Amsari	1000	120	100	13	30	14.3	71.4
Idjarane west	1100	80	80	16	30	9.4	46.8
Timimoune1	1550	100	75	13	30.2	8.6	42.9
Timimoune2	1800	800	50	14	30.2	49.8	249.2
Timimoune saouth	1500	95	68	17	30.3	10.3	51.3
Gourara 1	1400	1000	50	8	31.4	34.0	170
Gourara 2	1320	168	90	13	31.4	17.1	85.5
Gourara 3	1360	180	90	12	31.5	16.3	81.6
Total						1032.6	5162.8

## Results

The research and analytical work performed here led us to identify 12 suitable areas represented by deep saline confined and semi-confined aquifers (Table 4 and Fig. 1a). This analysis of the storage potential of the sedimentary basins in southwest Algeria for GSC allows us to highlight the attractive potential of the Ahnet–Gourara Basin (Fig. 1a). In fact, its final score of 0.92 % (Table 3) is greater than those of the other basins, and it is a priority basin for further investigations to identify specific sites and basins for CO_2_ injection.

The purpose of site characterization is to determine whether a site is suitable and safe for sequestration and to compile the data necessary for the permit application. The process includes geologic, geophysical, and engineering evaluations. Characterization is performed to obtain the geological and hydrological data needed to design the infrastructure, develop reservoir models, and design the monitoring program. In this phase of site development, a determination is made regarding whether the reservoir has adequate porosity, permeability, and continuity for long-term injection. In addition, the ability of overlying units to confine the injected CO_2_ and prevent vertical movement is also determined, including an evaluation of the presence of non-sealing faults and other potential pathways for migration. This analysis primarily focused on the geology (stratigraphy and structure) and geophysical data (seismic reflection and logging) for the characterization of potential reservoir-caprock systems.

Suitable areas (Ahnet and Gourara basins) are characterized by thick sediment, porous and permeable formations with saline water saturations that may exceed 97 %, and caprock with very low porosity that can act as an impermeable waterproof covering; the various characteristics of the reservoirs are listed in Table 4. Figure 4b shows the variation of water saturation in the Ahnet–Gourara basins.

Based on the work of Bachu (2003), twelve potential areas were considered to be moderately hot basins with geothermals not exceeding 32 °C/km, lying at depths of 1100 and 2200 m with temperatures and pressures sufficient to ensure the supercritical state and buoyancy of the injected CO_2_. Most of these saline aquifers are favorable for storing CO_2_ because of the thick Cambro–Ordovician layers of quartzitic sandstone with matrices of laminated clay intercalation and dispersed clay. The caprock has a minimum thickness of 200 m and is composed primarily of Silurian clay, which completely seals the potential reservoir. This initial assessment of the GSC capacity in Algerian deep saline aquifers encountered difficulties related to the storage efficiency factor, E_salin_, and the potential storage capacity was calculated assuming that 0.54 or 5.4 % of the total pore volume could be filled or saturated (NETL 2010). Our calculations provide a very conservative estimation of the effective capacity of the determined areas’ pro-sequestration of CO_2_ of approximately 1 GT or 5 Gt (E_salin_ = 1 and 5 %, respectively; Table 4).

Below, we present the main characteristics of the most promising area for CCS in Algeria, the Ahnet Basin. We noted a significant number of structures of varying sizes via the controlled mapping of the surface outcrops. Analyzing the seismic maps reveals a degree of intense structuring in this area, which contains an interesting mix of prospects and structurally complex types. The Ahnet Basin differs from other areas in the Saharan platform by its degree of intense structuring linked to the evolutionary history of the West African craton junction, which is thought to have been stable for approximately 2 billion years. The East African craton is considered mobile and cratonic because of the Pan-African orogeny (approximately 550–600 million years ago). The Ahnet Basin is related to a joint area of these two cratons. Their collision created brittle tectonic activity of the substratum level and is probably Paleozoic in age (Fabre et al. 1996). This old tectonic activity occurred during the following phases:

- Taconic (late Ordovician);
- Caledonian (Late Silurian beginning Devonian (Siegenien);
- Hercynian, the most important phase (late Permian); and
- Austria, an essentially post-Hercynian compression phase (Upper Cretaceous).

The current structure was primarily formed during the Hercynian orogeny, which completely modeled this basin (e.g., faults, gouge zones, anticlinal structures, and intense erosion). The Austrian phase wrinkling caused replays in which slip formed drive folds along preferential axes.

Moreover, this basin was also strongly influenced by tectonics linked to Hoggar and characterized by the presence of structural trends in the sub-meridian direction attached to the extension; the deformations north of the base are typical in the Hoggar.

Below, we present the main characteristics of two of the most promising areas for the application of CCS in Algeria. These two areas are located in southwest Algeria and in the onshore region and have been named “Tidikelt North” and “Akabli 02”, respectively (Fig. 1a).

### Tidikelt North

**Fig. 6:**
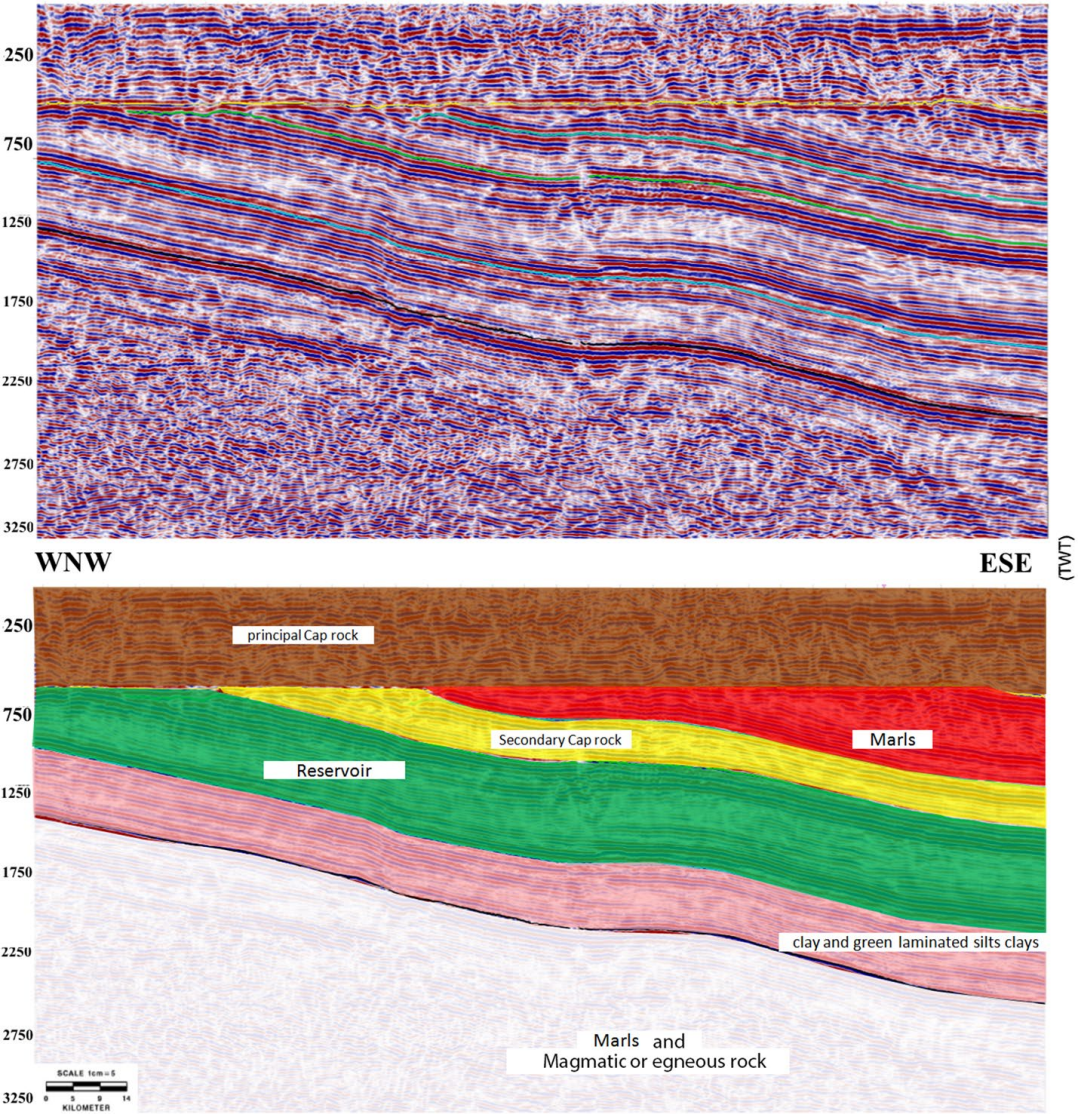
Example of a seismic profile collected across “Tidikelt North” and its interpretation. The *line* location is shown in Fig. 1a

Tidikelt North is located in the Ahnet Basin, specifically in the Ouallen Sub-Basin, which is bound to the east and west by large collisions and is filled by a thick sedimentary series of more than 7000 m, including lower outcrops to the east and west that represent one of the main structural elements in the field. Drilling and seismic studies established in the region show purple surmounted by a series of Cambro–Ordovician or infra-tasilienne unconformities located at a depth of 1200 m with a thickness of 700 m and an oblique stratification (Fig. 6).

The potential reservoir formations suggest an eolian deposit consisting of sands and silty sands from the Precambrian. These deposits have been interpreted as the bottom of the sub-basin. The reservoir is locally more than 500 m thick with an effective thickness exceeding 250 m, as recorded by several drill holes. Its sand layers are often saturated with salt water, as indicated by the spontaneous potential and resistivity logs and an analysis of the formation water that revealed salt saturation of 120–180 g/L. All wells drilled in this region have logging and the petrophysics of the logging recordings or the core, and a porosity of 18 % was assessed via a combination of different logs (namely, the sonic, neutron, and density logs), other photoelectric factors, and cores.

**Fig. 7:**
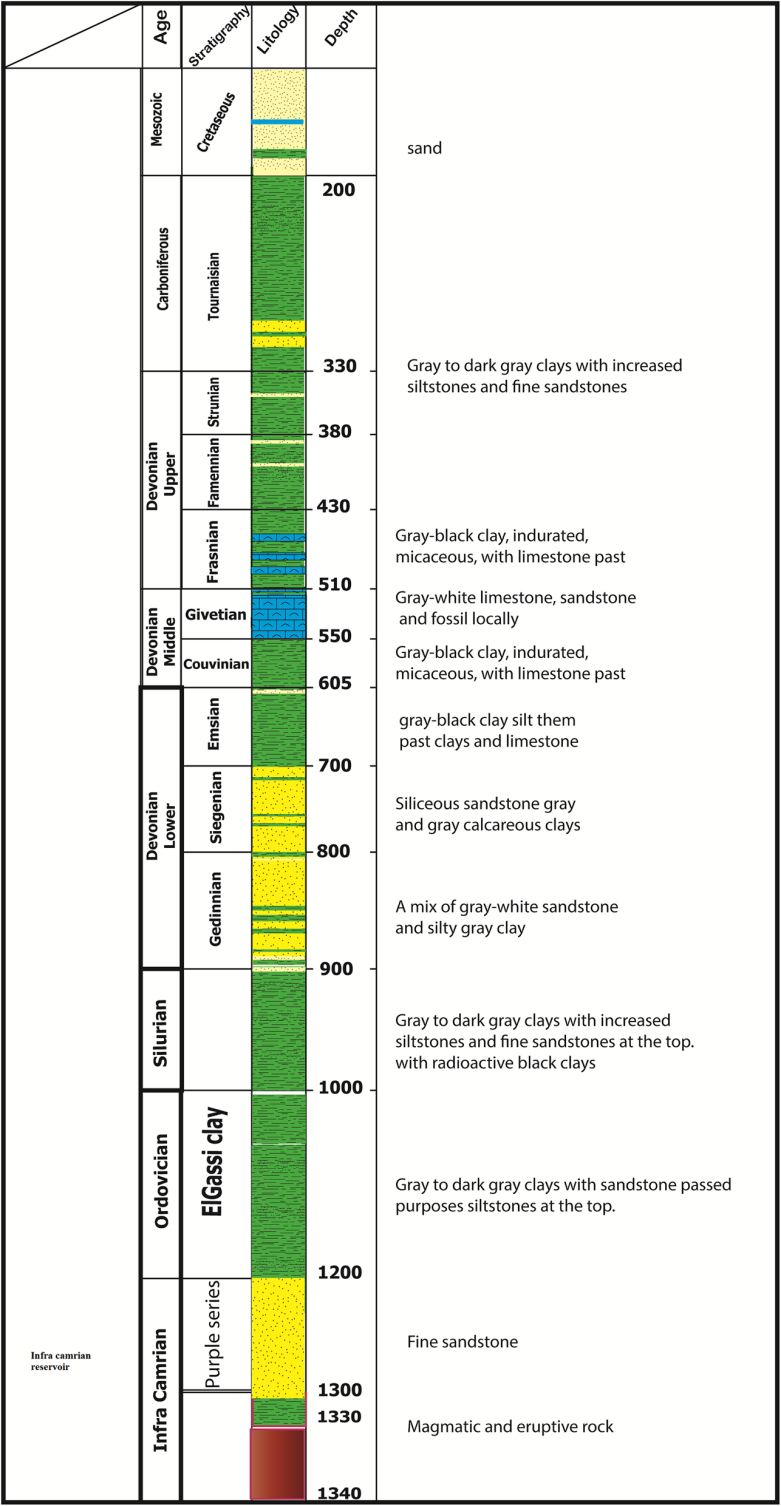
Schematic representation of a “composite log” of one of the boreholes in “Tidikelt North”

From seismo-stratigraphic and seismic perspectives, these deposits are represented by a considerable amplitude and subparallel and continuous reflections and overcome a Cambro–Ordovician unconformity or infra-tasilienne unconformity. Additionally, a thick, 1200-m sedimentary series of shales of Ordovician and, specifically, El Gassi Clay play the role of caprock in addition to the Silurian shales, which represent the regional coverage for the Cambro–Ordovician reservoirs. To the west of the reservoir series based on infra-Cambrian formations of clay and green finely laminated silts clays often containing dropped pebbles (dropstones), the depositional environment corresponds to a sea or lake environment (Fig. 7).

With an area of approximately 1150 km^2^, this structure is one of the most promising sites for CO_2_ sequestration. Additionally, because of its density of approximately 920 kg CO_2_/m^3^ (Fig. 3b), this site could store over 476 Mt of CO_2_.

### Akabli 02

Akabli 02 is located in the region of Akabli in northwest Ahnet. The current geometry of the region is marked by various superimposed major structural axes resulting from a complex polyphase history. The N–S and NW–SE directions approximately follow the collisional frontline between the rigid Precambrian craton in West Africa and the bulk of East Africa, which has experienced terrane accretion and additional distortion. The NW–SE direction controls the sedimentation from the Silurian with the opening of the basin to the north and marks the structuring of the region because of the reversal of the paroxysmal movements from the Hercynian to the late Carboniferous. Finally, the E–W and NE–SW directions experienced transgressive movements in the Hercynian.

The area subsided during the Mesozoic, and the tectonic impact of the Cretaceous and Tertiary was relatively limited at the basin scale. Structural traps, which primarily formed during the Carboniferous, are usually associated with large reverse faults with depressions of up to several hundred meters. They have high amplitudes but moderates size (several kilometers of expansion along the structure’s axis).

Akabli 02 is a high-amplitude anticline with a WSW-ENE axis approximately 380 km^2^ at its structural closure at the roof of the Ordovician (1165 m below sea level). This structure is located in a relay zone between two reverse faults trending sinistral N70° and showing decoupling.

**Fig. 8:**
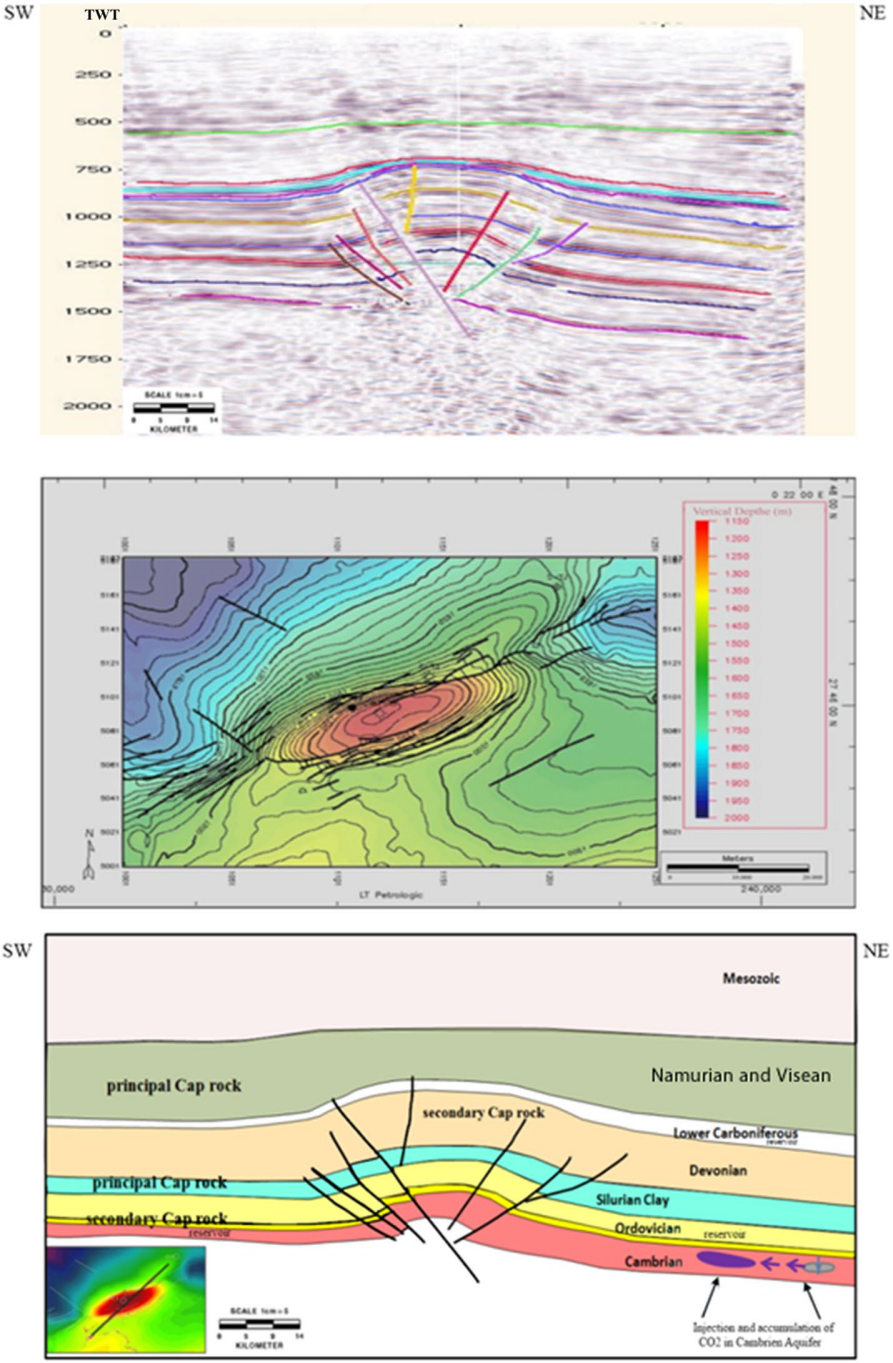
Example of a seismic profile collected across “Akabli 02” and its interpretation. The *line* location is shown in Fig. 1a

This structure is affected by numerous faults related to the tectonic style and primarily the decoupling zone. It is pinched between two thrust faults with strong subsidence and, likely, a slight sinistral strike slip motion (Fig. 8). Its establishment is associated with a principal stress oriented at N120°, which corresponds to the direction of the Hercynian compression observed in the region.

Regional data show that the Cambrian deposits are regular and very uniform over large distances. Cambrian sands were deposited in a continental environment as infilling and were amalgamated into braids. They are the main structure in the reservoir.

More resent episodes of an estuarine environment are also revealed in the core drilling intervals and are reflected and increased relative to the thinner clay sediment. This depositional environment explains the lack of contrast and outstanding figures in the logs, which make them difficult to correlate.

The homogeneity of the reservoir’s mode of filling is reflected by the absence of reliable subdivision within the Cambrian and significant extension to the fluid flow barrier. However, the top of the Cambrian unit is identifiable based on the responses of the porosity, particularly the density and sonic logs. The Cambrian unit is the main reservoir. Regional data show that this reservoir was deposited relatively uniformly over large areas with large thicknesses. Thus, this reservoir is modeled with a thickness of 250 m, a uniform structure, and a porosity of 15 %. Other formations found during drilling were taken as secondary reservoirs and were evaluated by means of logs, which demonstrated the existence of salt water.

Ordovician Units IV & II are primarily sandstone with past micro conglomeratic clays. This reservoir is the main gas reservoir in the Ahnet Basin. The porosity exceeds 15 % in the southern edge of the region and is approximately 6–8 % in the central part.

The Tournaisian unit contains fine sandstone with glauconite and bioclastic in the form of 50-m marine bars. The reservoir’s characteristics are generally good. The porosity exceeds 15 %, and the permeability exceeds 100 mD; however, it is not considered a favorable reservoir because of its depth (less than 800 m).

The Strunien unit often corresponds to sandstones in communication with the Tournaisian sandstones and is essentially an aquifer. The average porosity exceeds 20 % at the top and decreases with depth. The permeability rarely exceeds 100 Md. However, this reservoir is not considered favorable because of its depth.

The Gedinnian unit corresponds to sandstone bar-type deposits of bioturbated sandstone and clay. The porosity ranges between 8 and 16 %, and its permeability rarely exceeds 1 mD. The thickness is variable and sometimes exceeds 95 m. Logs indicate a column of 82 m with porosity as high as 14 %.

The Givetian unit consists of bioclastic calcareous sediments alternating with clay and sandstone. The thickness is variable and sometimes exceeds 50 m. Logs indicate a column of 48 m with porosity as high as 25 %.

**Fig. 9:**
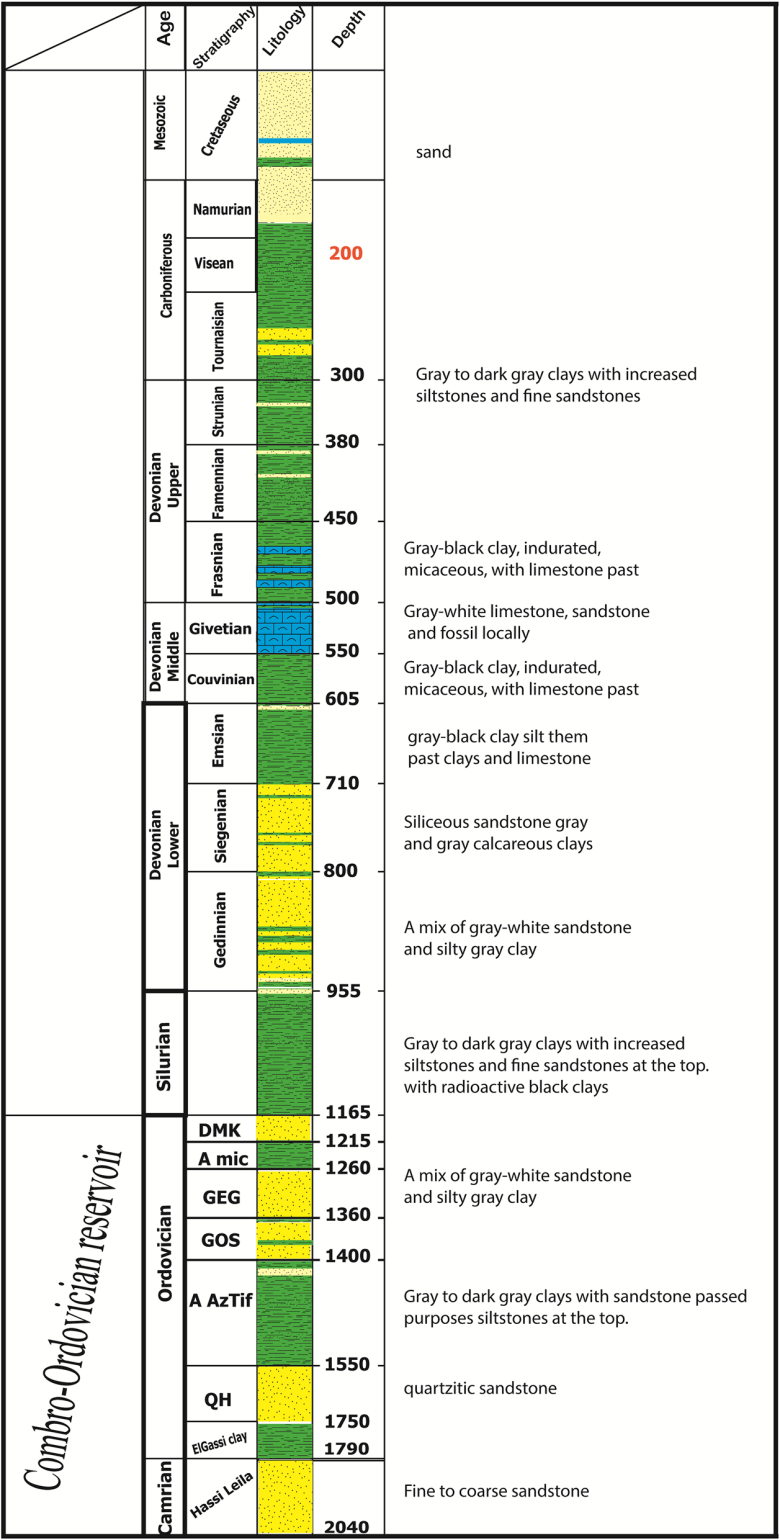
Schematic representation of a “composite log” of one of the boreholes in “Akabli 02”

Coverage of these reservoirs is provided by Silurian clays, which are regionally well developed and provide a good coverage of the Cambro–Ordovician reservoirs. The base of these clays is highly radioactive with abnormally high pressures, increasing their coverage effectiveness. The Tournai reservoir sandstones are covered by Tournaisian and Namurian clays, and the Middle and Upper Devonian clays provide a caprock for the Gedinnian and Siegenien sandstones (Fig. 9).

**Fig. 10:**
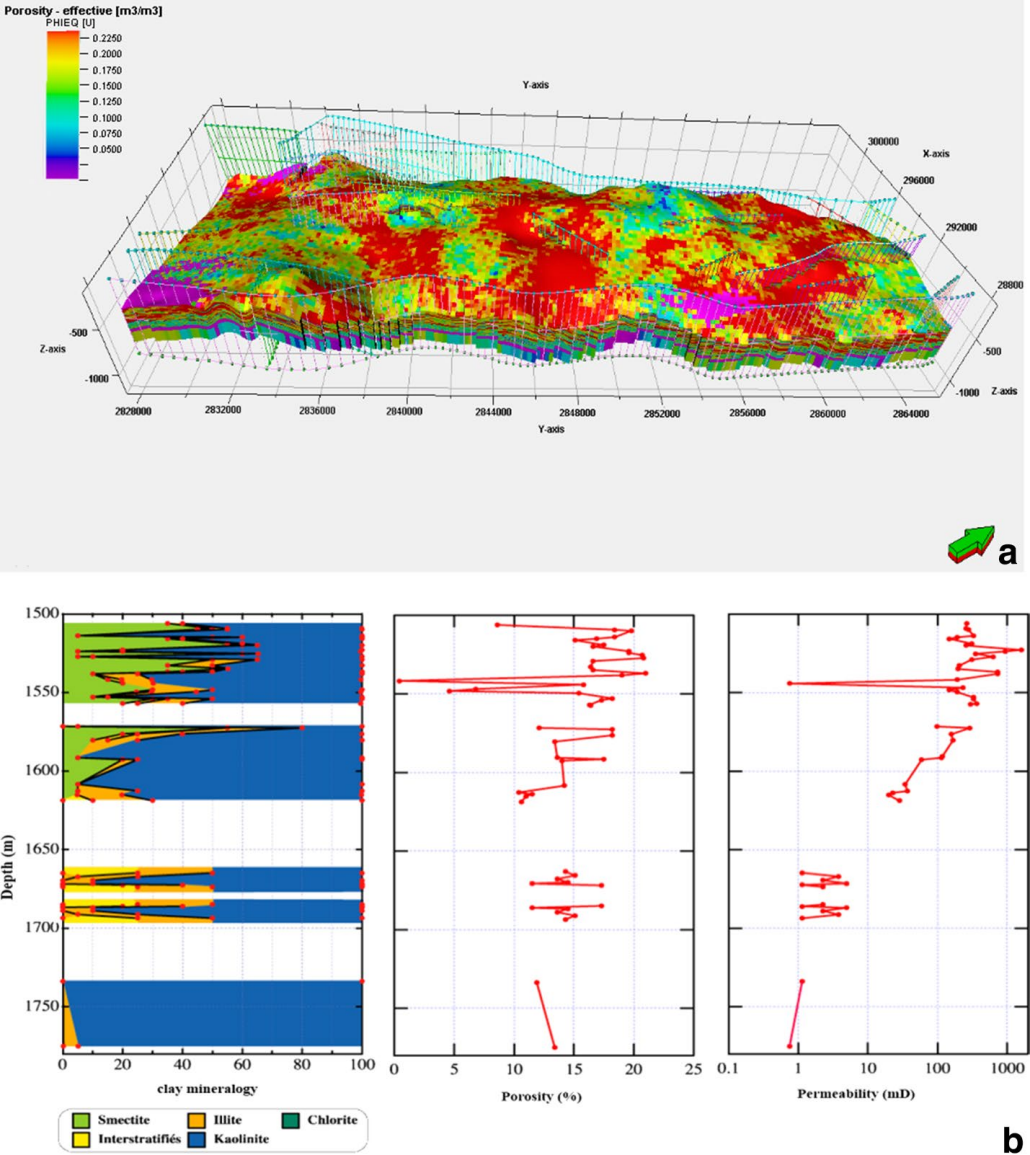
**a** Porositymodel in Akablie 02. **b** Mineralogical and petrophysical variation depending on the depth in Akablie 02

The different relationships of the porosity/permeability and Cortege clay are shown in Fig. 10. With an area of almost 380 km^2^, this structure is one of the most promising sites for CO_2_ sequestration with a density of approximately 960 kg CO_2_/m^3^ (Fig. 3b) and could store over 345 Mt.

## Discussion and conclusions

Analyzing the potential of sedimentary basins in southwest Algeria for GSC highlighted the interesting potential of the Ahnet–Gourara Basin. Indeed, its final score stands out from those of other basins, and thus, this basin is prioritized for further studies to determine and identify specific sites and reservoirs for CO_2_ injection. Our assessment of the total CO_2_-storage capacity in the Ahnet–Gourara Basin allowed for the identification of 12 suitable areas that could potentially store Algeria’s annual CO_2_ emissions for the next 50 years. This value represents a very conservative estimate of the GSC capacity in the deep saline aquifers of the Ahnet–Gourara Basin because other promising reservoirs could be found in areas where data are not currently available, such as the Bechar and Tindouf Ponds, which are being explored.

Despite these uncertainties, this is the first report of the estimated GSC potential and storage capacity of deep saline aquifers in Algeria. This study demonstrates that CO_2_ storage in deep saline aquifers is a viable option for Algeria. However, because of the lack of several types of data, such as the physical and mechanical properties of the reservoir caprock, our assessment of the storage capacity in Algeria’s deep saline aquifers is far from complete. In fact, this study represents the starting point for a more detailed future analysis.

Our study also suggests that all countries geologically characterized by deep saline formations may contain suitable reservoirs for GSC. These results are particularly interesting given the objectives set by international agreements, including the Kyoto Protocol. Indeed, the widespread use of CCS will be necessary in both developed countries and emerging countries.
